# Supplementary material for: De novo virulence feature discovery and risk assessment in Klebsiella pneumoniae based on microbial genome vectorization
Source: Commun Biol. 2025 Apr 17;8:623. doi: 10.1038/s42003-025-07678-9 (PMC12006392; doi:10.1038/s42003-025-07678-9)
Supplement: Supplementary file 1 — Supplementary Information [file 42003_2025_7678_MOESM1_ESM.pdf]

Table S1: FGP IPR Domain Summary

IPR Type	# Distinct IPR	# Distinct Protein
ACTIVE_SITE	129	969,187
BINDING_SITE	73	861,228
CONSERVED_SITE	606	6,301,098
DOMAIN	6,827	29,013,089
FAMILY	14,591	24,771,707
HOMOLOGOUS_SUPERFAMILY	2,569	30,900,236
PTM	12	250,448
REPEAT	236	912,019

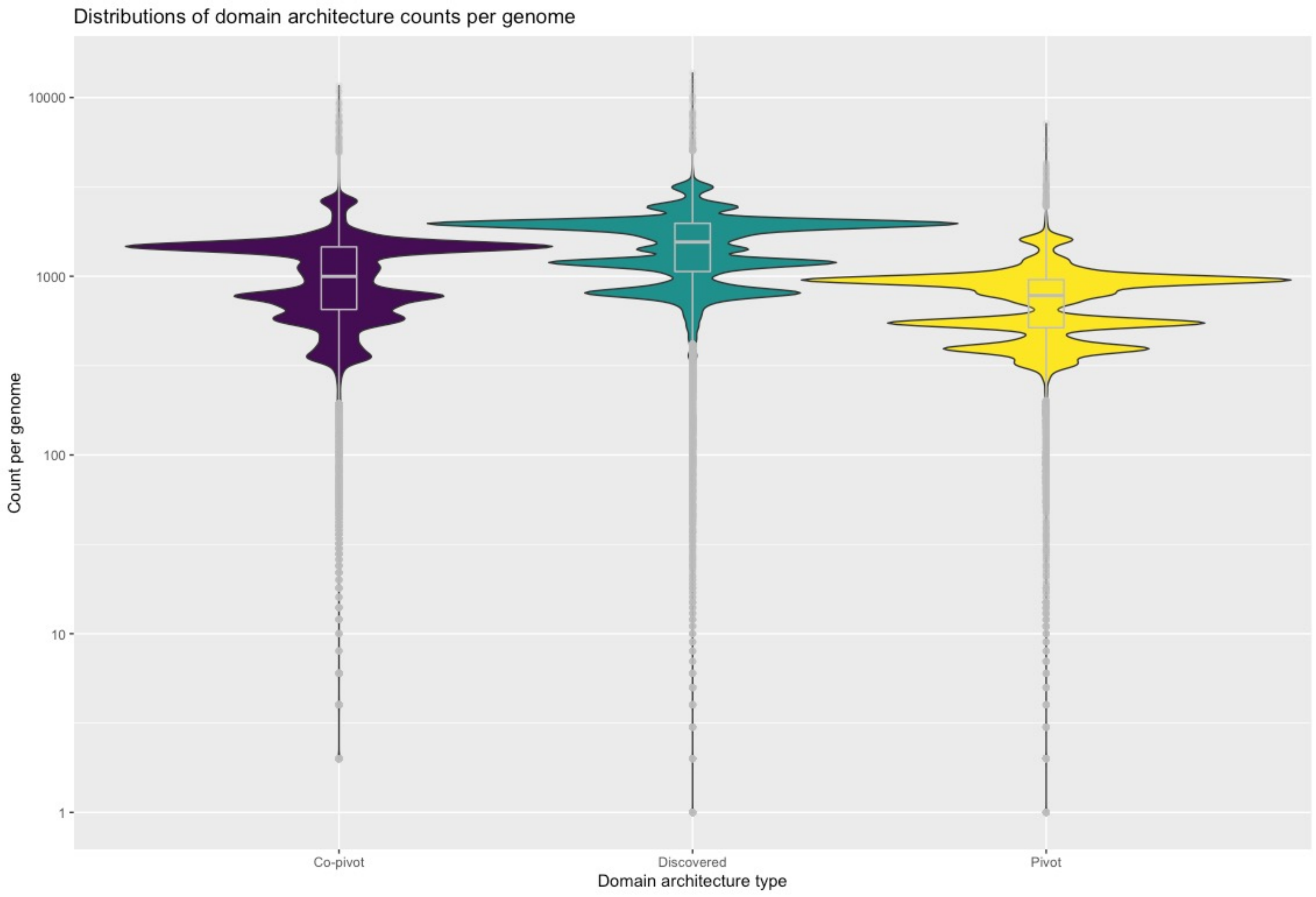

Figure S1: Distribution of domain architecture types per genome. The total count of unique co-pivot, discovered, or pivot domain architectures in a given genome is plotted for the entire genome corpus analyzed.

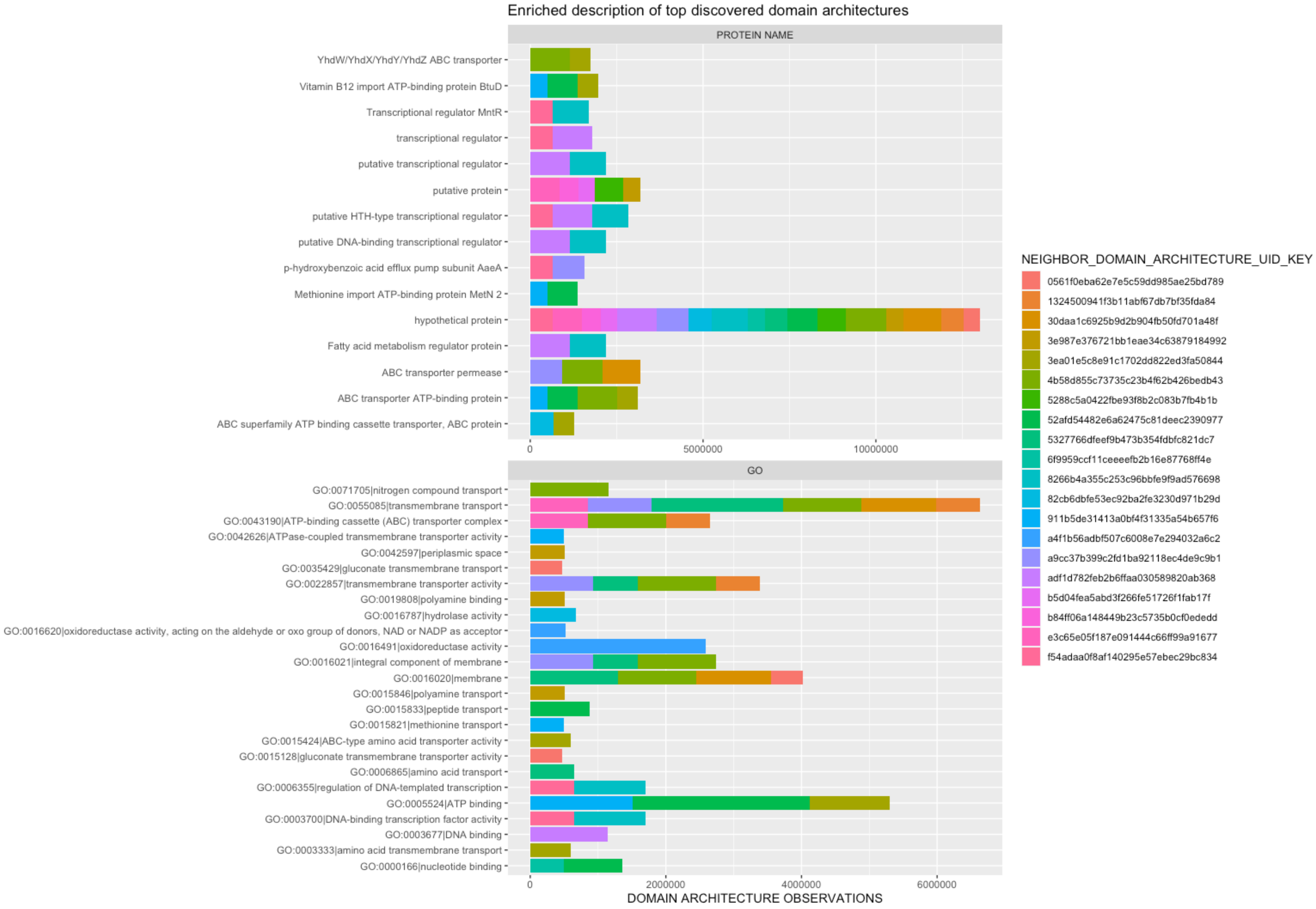

Figure S2: Top twenty discovered domains architectures (DA) by occurrence across all genera are described by the set of enriched protein names (protein names occurring for more than one top DA) in top panel and their respective GO terms (bottom panel).

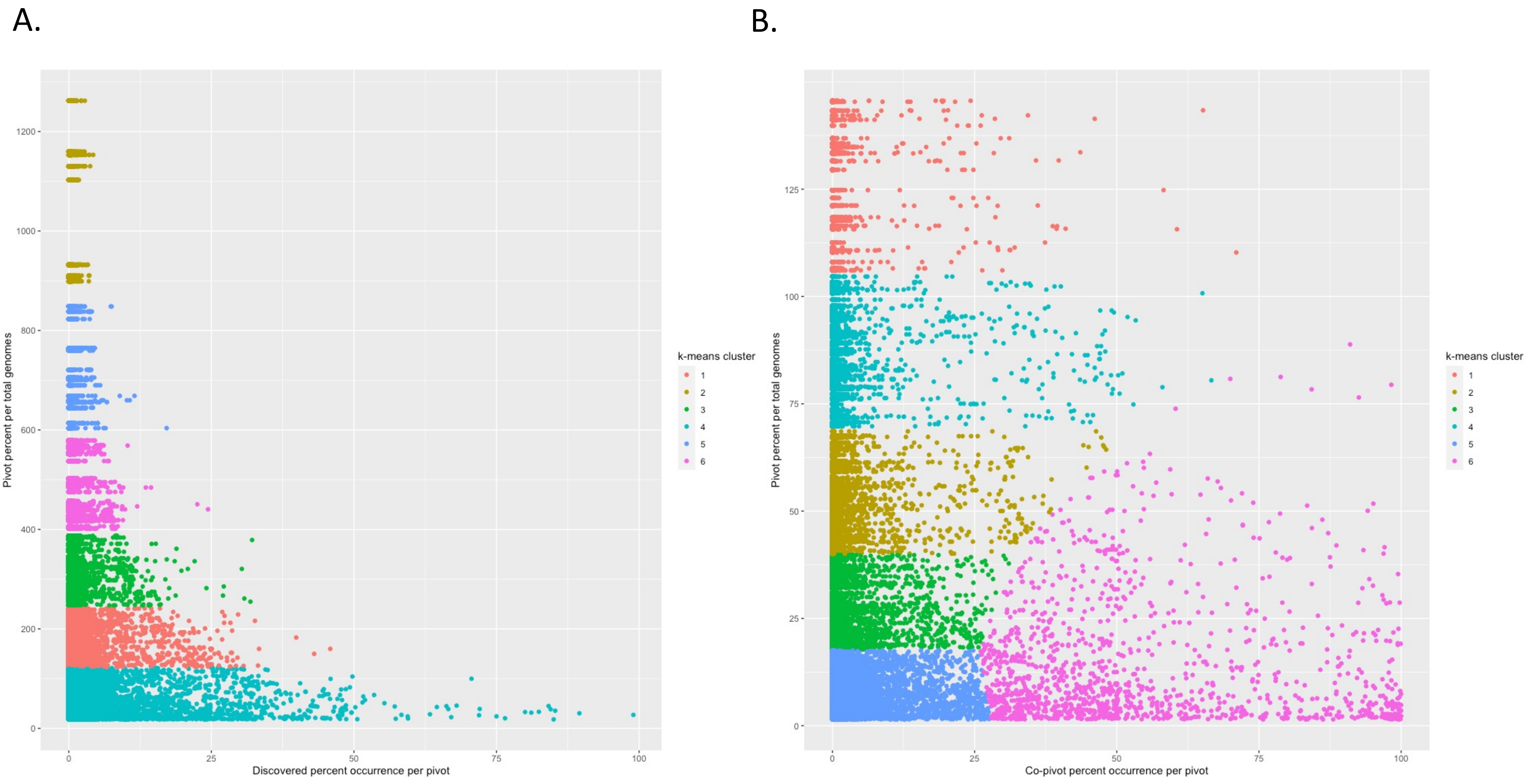

Figure S3: Occurrence of pivot:co-pivot and pivot:discovery domain architecture pairings across all genera are clustered using k-means. The percentage a pivot DA occurs across all genomes in a cohort is indicated in the y-axis as well as the percent that a discovered (A) or co-pivot (B) domain architecture occurs as its direct neighbor is shown.

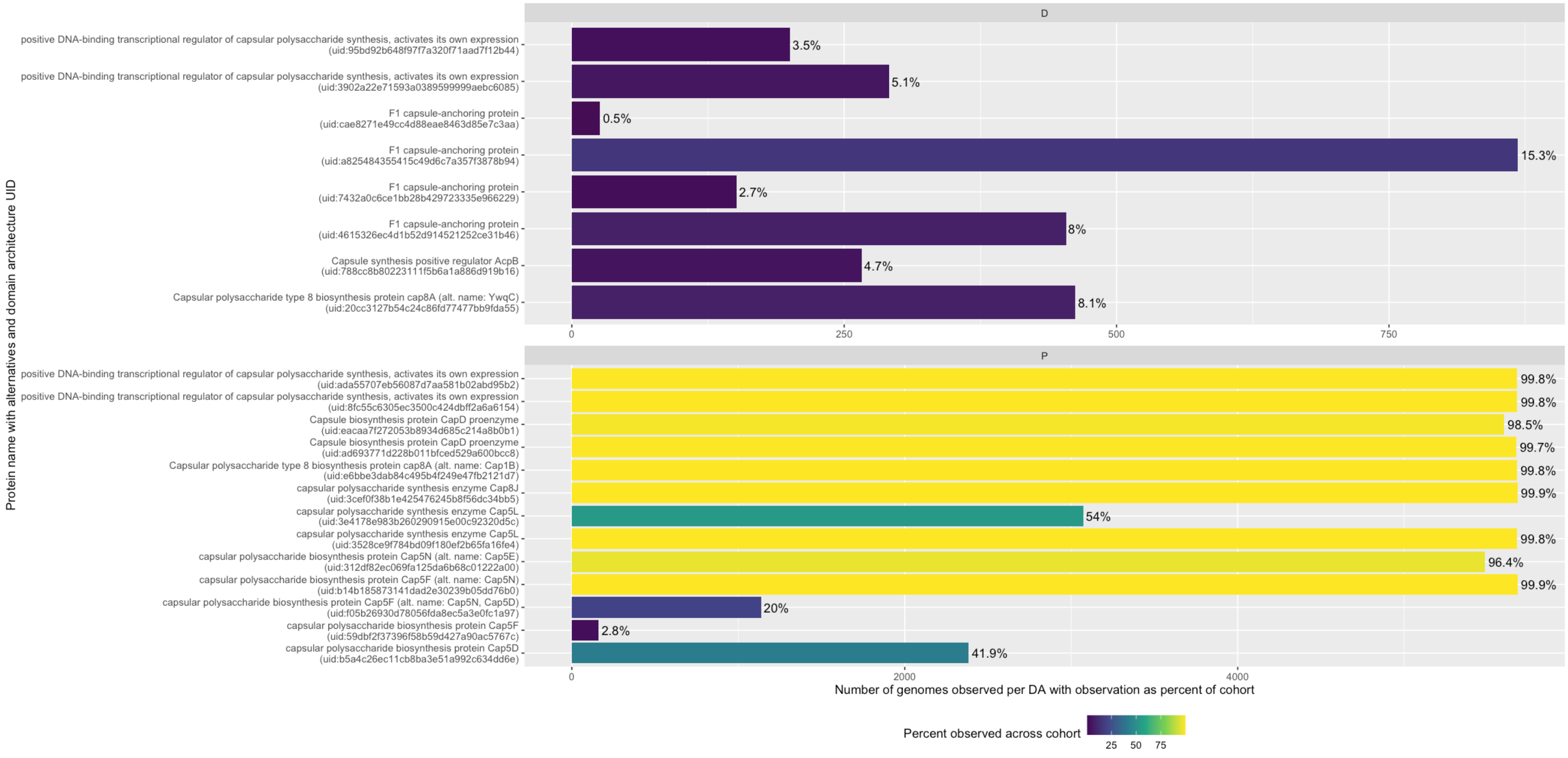

Figure S4: Assessment of capsular content in *Klebsiella* genome cohort for discovered (top) and pivot (bottom) DAs where protein name contains “capsule”, ”capsular”, or “capsule expression.” The count value is indicated by bar length and the respective percentage of each DA’s observation across the cohort is indicated at the end of the bar. Alternative protein names are provided in parentheses for each DA as appropriate.

Table S2: Virulence Category Definitions

Virulence Category	Definition
NOT_VIRULENT	Environmental and animal isolates--note that enviornmental isolates are not here differentiated by clinical or non-clinical, acknowledging clinical environmental isolates are likely human-associated and therefore may be at least as virulent as colonization
COLONIZATION	Rectal swab, skin, feces
LOW_VIRULENT	All clinical isolates from body sites or specimen sources that are from 'non-sterile body sites' (incl drainage, sputum, respiratory, wound, and urine)
HIGH_VIRULENT	Blood, aspirate, abscess, CSF, tissue
MISSING**	Missing, dash, empty, 'non-applicable', etc
AMBIGUOUS_CLINICAL**	Associated with healthcare but cannot differentiate virulence level--note that some body fluids appear here although they could be from even 'sterile body site', don't know how they were collected and whether indwelling drain may have become colonized
AMBIGUOUS_GENERAL**	Not well-associated with healthcare and cannot differentiate virulence level--note that isolates labled only by hospital name appear here
***Denotes categories excluded from model creation due to insufficient information.*	

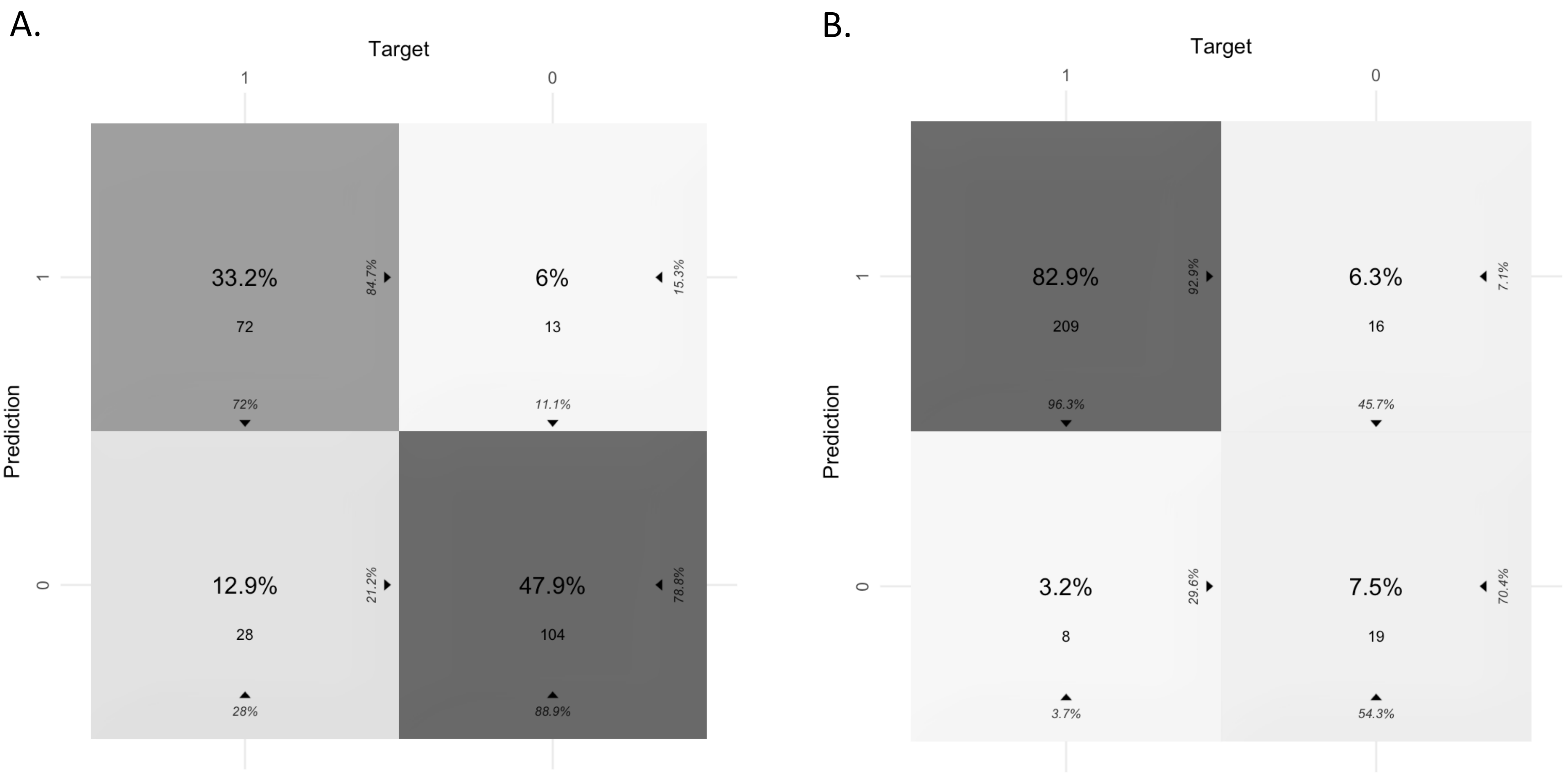

Figure S5: Confusion matrices are shown for the best performing models of each classification task: high versus low virulence (A) and virulent versus not virulent (B).

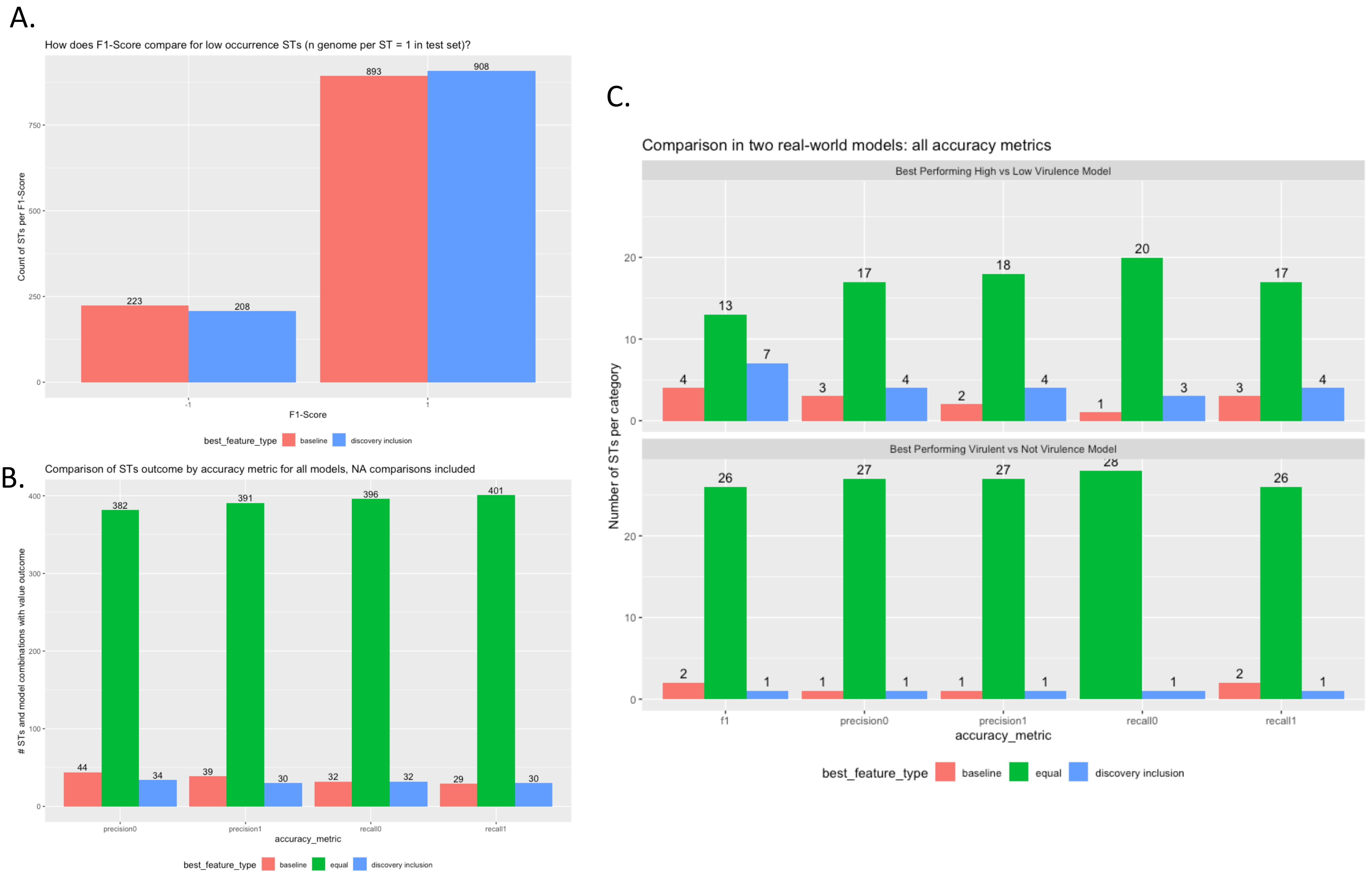

Figure S6: Error analysis by MLST are indicated for baseline and discovery inclusion feature sets. The F1-Score is compared for STs with one genome in the test set (A) where -1 is a representation of F1-Score = NaN. The precision and recall values for all other STs are evaluated for <u>all models</u> for which feature set is better performing or if the accuracy metrics indicate the feature sets yield equivalent results and the counts are shown (B). The best performing models of each classification task are shown in (C) with the feature type that yielded the highest accuracy.

Table S3: MLST Diversity by Virulence Category

VIRULENCE CATEGORY	# distinct MLST	# samples	MLST diversity per samples
NOT_VIRULENT	54	102	**0.53**
COLONIZATION	78	250	**0.31**
HIGH_VIRULENT	232	1089	**0.21**
LOW_VIRULENT	195	1361	**0.14**

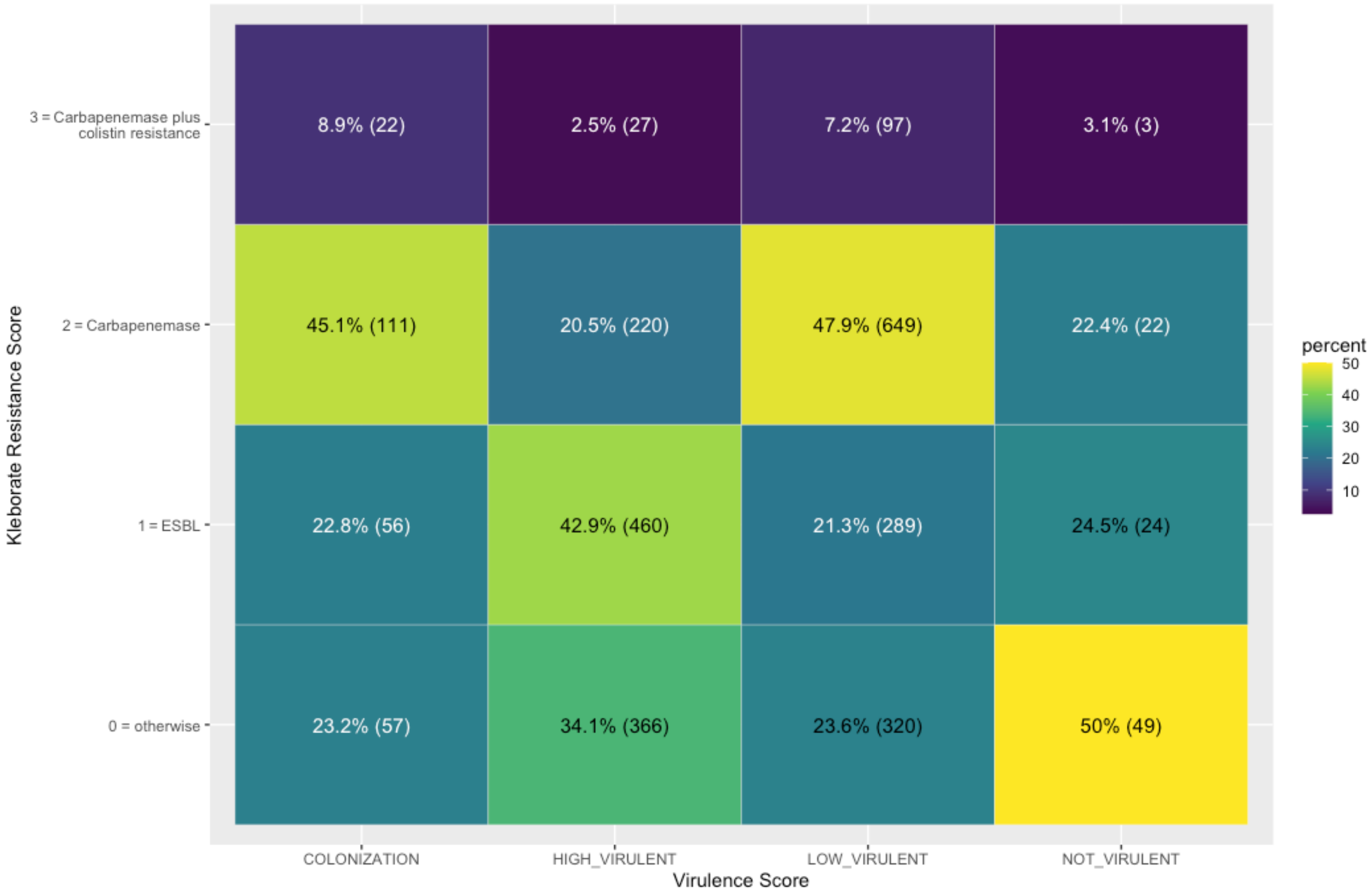

Figure S7: The virulence category for *Klebsiella* isolates is contrasted with the resistance score as identified from Kleborate. The percent of isolates per virulence category is shown (i.e. 45.1% of colonization isolates exhibited a Kleborate resistance score of 2) as well as the count of isolates per category set.

Table S4: VFDB IPR Summary

IPR Type	# Distinct IPR	# Distinct Protein
ACTIVE_SITE	30	556
BINDING_SITE	19	247
CONSERVED_SITE	81	2,781
DOMAIN	923	13,919
FAMILY	1,101	13,851
HOMOLOGOUS_SUPERFAMILY	541	12,971
PTM	5	675
REPEAT	59	830

Klebsiella

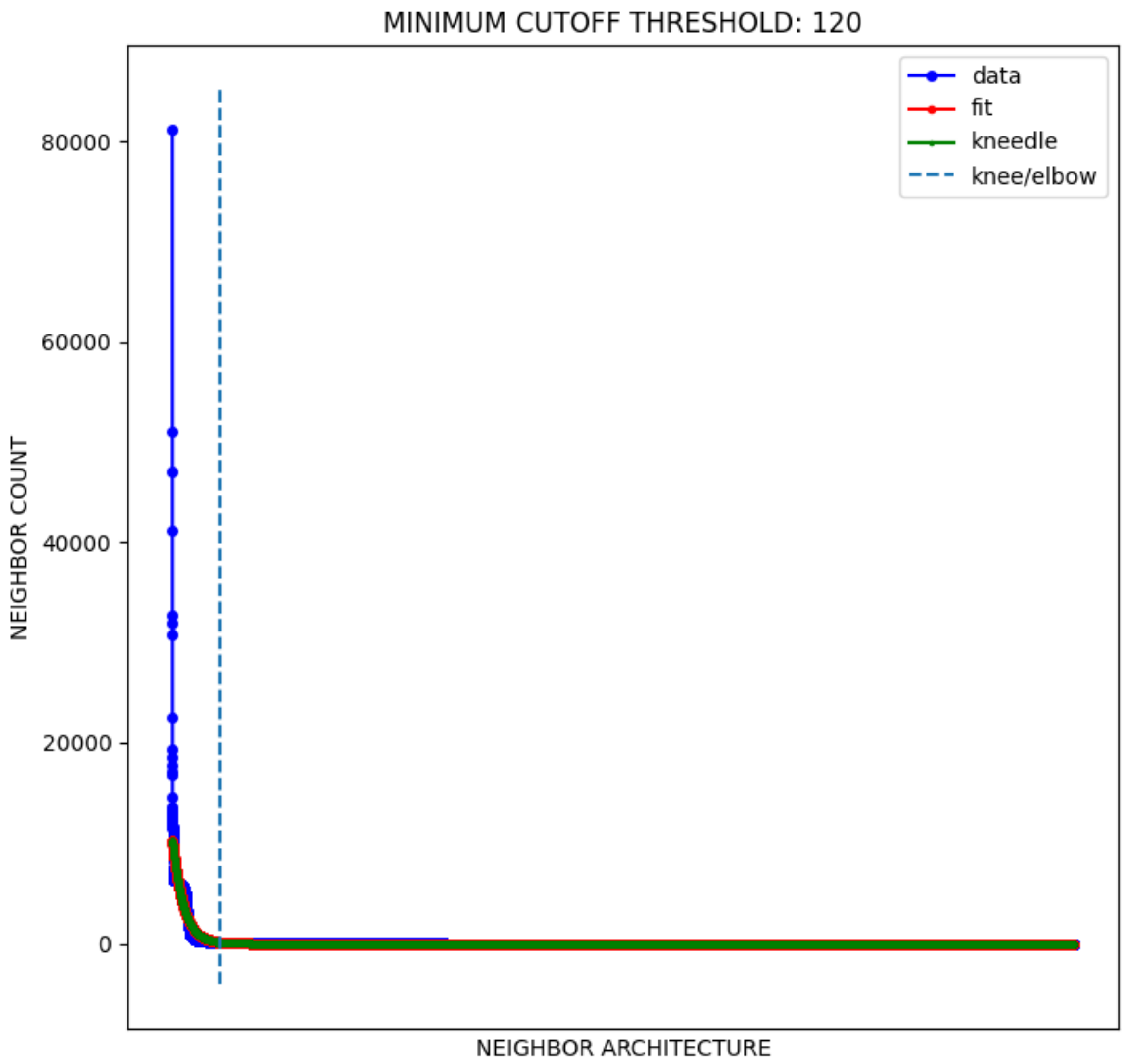

Figure S8: Example usage of computing minimum cutoff threshold for *Klebsiella* using Kneedle algorithm where neighbor count is observed count within that genera and neighbor architecture is the sorted domain architecture hash.

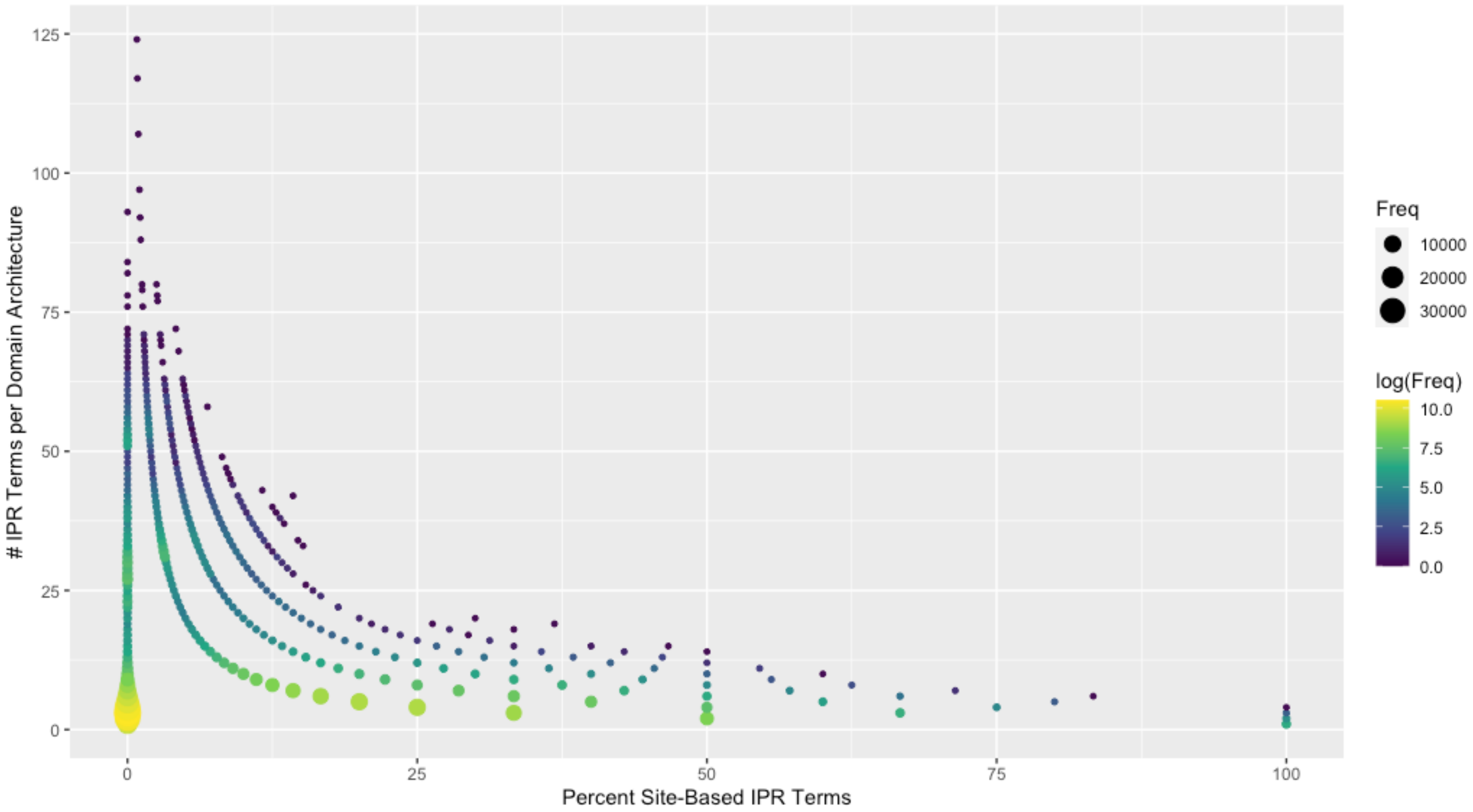

Figure S9: Assessment of Site-based IPR Terms in Domain Architectures. All domain architectures from seed data collection were assessed for the occurrence of site-based terms (ACTIVE SITE, BINDING SITE, CONSERVED SITE, PTM, or REPEAT) where the total number of IPR terms is contrasted against the percentage of those terms that are site-based. The frequency for that numerical combination is provided in color and circle size.

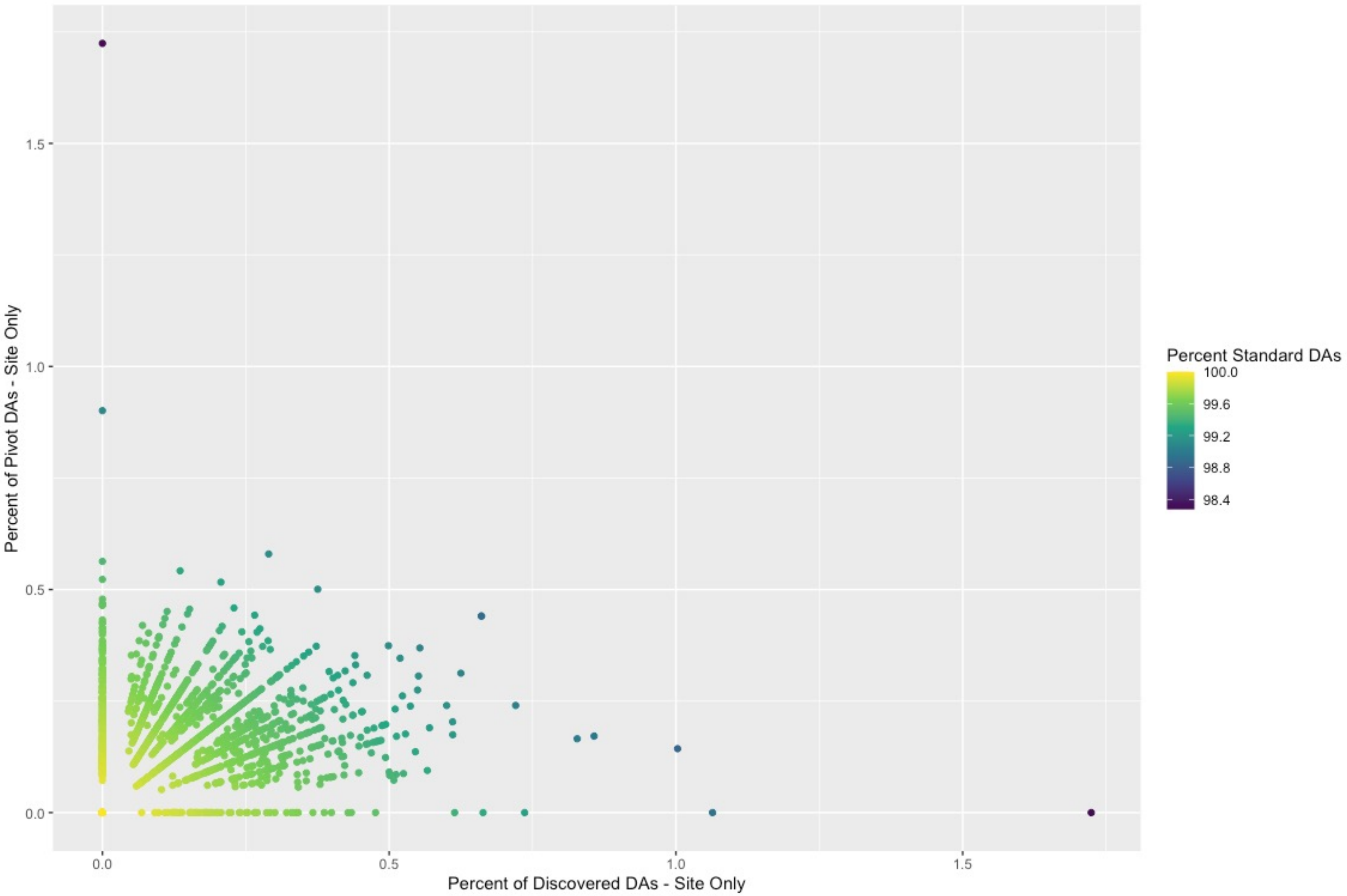

Figure S10: Assessment of Site-based IPR Terms in Discovered Domain Architectures. For each genera, the percentage of domain architectures comprised of all site-based terms is contrasted for discovered DAs and pivot DAs. The percentage of non-site-based terms i.e. standard intermixed domains architectures is indicated in color. Domain architectures included in this plot are those above the thresholds applied in kneedle analysis.
