# Supplementary material for: De novo virulence feature discovery and risk assessment in Klebsiella pneumoniae based on microbial genome vectorization
Source: Commun Biol. 2025 Apr 17;8:623. doi: 10.1038/s42003-025-07678-9 (PMC12006392; doi:10.1038/s42003-025-07678-9)
Supplement: Supplementary file 4 — Description of Additional Supplementary File [file 42003_2025_7678_MOESM4_ESM.pdf]

# Description of additional supplementary file

File name: Supplementary File S1
Description: number-features-dropped-discovery-inclusion.csv
Number of features dropped from discovery inclusion model permutations

File name: Supplementary-File-S2
Description: -stacked-unique-features-per-class.txt
Unique features (domain architectures) per class
