# Supplementary material for: De novo virulence feature discovery and risk assessment in Klebsiella pneumoniae based on microbial genome vectorization
Source: Commun Biol. 2025 Apr 17;8:623. doi: 10.1038/s42003-025-07678-9 (PMC12006392; doi:10.1038/s42003-025-07678-9)
Supplement: Supplementary file 5 — Reporting Summary [file 42003_2025_7678_MOESM5_ESM.pdf]

nature portfolio

Corresponding author(s): Beck, Kristen L. COMMSBIO-24-3045-A

Last updated by author(s): Jan 10, 2025

Reporting Summary

Nature Portfolio wishes to improve the reproducibility of the work that we publish. This form provides structure for consistency and transparency in reporting. For further information on Nature Portfolio policies, see our Editorial Policies and the Editorial Policy Checklist.

Statistics

For all statistical analyses, confirm that the following items are present in the figure legend, table legend, main text, or Methods section.

n/a	Confirmed	
☐	☒	The exact sample size (*n*) for each experimental group/condition, given as a discrete number and unit of measurement
☒	☐	A statement on whether measurements were taken from distinct samples or whether the same sample was measured repeatedly
☐	☒	The statistical test(s) used AND whether they are one- or two-sided<br>*Only common tests should be described solely by name; describe more complex techniques in the Methods section.*
☒	☐	A description of all covariates tested
☐	☒	A description of any assumptions or corrections, such as tests of normality and adjustment for multiple comparisons
☐	☒	A full description of the statistical parameters including central tendency (e.g. means) or other basic estimates (e.g. regression coefficient) AND variation (e.g. standard deviation) or associated estimates of uncertainty (e.g. confidence intervals)
☒	☐	For null hypothesis testing, the test statistic (e.g. *F*, *t*, *r*) with confidence intervals, effect sizes, degrees of freedom and *P* value noted<br>*Give P values as exact values whenever suitable.*
☒	☐	For Bayesian analysis, information on the choice of priors and Markov chain Monte Carlo settings
☒	☐	For hierarchical and complex designs, identification of the appropriate level for tests and full reporting of outcomes
☒	☐	Estimates of effect sizes (e.g. Cohen's *d*, Pearson's *r*), indicating how they were calculated

Our web collection on statistics for biologists contains articles on many of the points above.

Software and code

Policy information about availability of computer code

Data collection	Please see methods sections for extensive list especially Methods Section 4.1, 4.3, 4.8.1, and 4.8.2
Data analysis	Since this manuscript describes a computationally focused method, there is an extensive list of tools used and custom code developed and open sourced as part of this method. Please see the Methods Section for the most comprehensive list.

For manuscripts utilizing custom algorithms or software that are central to the research but not yet described in published literature, software must be made available to editors and reviewers. We strongly encourage code deposition in a community repository (e.g. GitHub). See the Nature Portfolio guidelines for submitting code & software for further information.

Data

Policy information about availability of data

All manuscripts must include a data availability statement. This statement should provide the following information, where applicable:

- Accession codes, unique identifiers, or web links for publicly available datasets
- A description of any restrictions on data availability
- For clinical datasets or third party data, please ensure that the statement adheres to our policy

Predicted virulence discoveries for all genera have been deposited to PrecisionFDA as a Spark warehouse \url{https://precision.fda.gov/home/assets/file-Gj2qKX00ZqJppv3JjxPFxJkG-1}. From the \textit{Klebsiella} virulence prediction use case, the feature tables, models, relevant metadata, and classification outputs

have also been open-sourced to PrecisionFDA \url{https://precision.fda.gov/home/assets/file-Gj87zv00ZqJQ36gqppFz9QQF-1}. Please see GitHub for complete documentation of data tables \url{https://github.com/IBM/omxware-getting-started/tree/master/Virulence-Analysis/methodology}.

Research involving human participants, their data, or biological material

Policy information about studies with human participants or human data. See also policy information about sex, gender (identity/presentation), and sexual orientation and race, ethnicity and racism.

Reporting on sex and gender	NA
Reporting on race, ethnicity, or other socially relevant groupings	NA
Population characteristics	NA
Recruitment	NA
Ethics oversight	NA

Note that full information on the approval of the study protocol must also be provided in the manuscript.

Field-specific reporting

Please select the one below that is the best fit for your research. If you are not sure, read the appropriate sections before making your selection.

☒ Life sciences ☐ Behavioural & social sciences ☐ Ecological, evolutionary & environmental sciences

For a reference copy of the document with all sections, see nature.com/documents/nr-reporting-summary-flat.pdf

Life sciences study design

All studies must disclose on these points even when the disclosure is negative.

Sample size	All bacterial genomic data was collected from NCBI Genbank and SRA and used at the time of the study. For the Klebsiella use case, all Klebsiella data available from the aforementioned sources was used.
Data exclusions	Data exclusions are detailed in Methods Section 4.8.1 relating to completeness of bacterial genome metadata and Methods Section 4.1 relating annotated protein domain architectures.
Replication	In the Klebsiella use case, the test and train genome sets were constructed using a fixed random seed which allows for reproducibility in the construction of those sets. Additionally, hyperparameter tuning was performed for all models using Bayes Search Cross-Validation in the Python scikit-optimize package with 5-fold cross-validation and up to 100 iterations of Bayes Search.
Randomization	See above for machine learning use case. Allocation of experimental groups was not part of our analysis workstream.
Blinding	In the Klebsiella use case, virulence category definitions were constructed without knowledge of the isolation source distribution or attribution per genome. No other blinding was necessary in other analysis streams.

Reporting for specific materials, systems and methods

We require information from authors about some types of materials, experimental systems and methods used in many studies. Here, indicate whether each material, system or method listed is relevant to your study. If you are not sure if a list item applies to your research, read the appropriate section before selecting a response.

Materials & experimental systems

n/a	Involved in the study	
☒	☐	Antibodies
☒	☐	Eukaryotic cell lines
☒	☐	Palaeontology and archaeology
☒	☐	Animals and other organisms
☒	☐	Clinical data
☒	☐	Dual use research of concern
☒	☐	Plants

Methods

n/a	Involved in the study	
☒	☐	ChIP-seq
☒	☐	Flow cytometry
☒	☐	MRI-based neuroimaging

Plants

Seed stocks	NA
Novel plant genotypes	NA
Authentication	NA
